# Nanoparticles as elicitors and harvesters of economically important secondary metabolites in higher plants: A review

**DOI:** 10.1049/nbt2.12005

**Published:** 2021-02-19

**Authors:** Sanchaita Lala

**Affiliations:** ^1^ Department of Botany Sarsuna College University of Calcutta Kolkata West Bengal India

## Abstract

Nanoparticles possess some unique properties which improve their biochemical reactivity. Plants, due to their stationary nature, are constantly exposed to nanoparticles present in the environment, which act as abiotic stress agents at sub‐toxic concentrations and phytotoxic agents at higher concentrations. In general, nanoparticles exert their toxicological effect by the generation of reactive oxygen species to which plants respond by activating both enzymatic and non‐enzymatic anti‐oxidant defence mechanisms. One important manifestation of the defence response is the increased or *de novo* biosynthesis of secondary metabolites, many of which have commercial application. The present review extensively summarizes current knowledge about the application of different metallic, non‐metallic and carbon‐based nanoparticles as elicitors of economically important secondary metabolites in different plants, both *in vivo* and *in vitro*. Elicitation of secondary metabolites with nanoparticles in plant cultures, including hairy root cultures, is discussed. Another emergent technology is the ligand‐harvesting of secondary metabolites using surface‐functionalized nanoparticles, which is also mentioned. A brief explanation of the mechanism of action of nanoparticles on plant secondary metabolism is included. Optimum conditions and parameters to be evaluated and standardized for the successful commercial exploitation of this technology are also mentioned.

## 1 INTRODUCTION

Nanotechnology is probably the greatest technological revolution to impact the world in the new millennium. It is a technology defined by size, in the sense that it encompasses all technology within the nanometre size range. Of these, nanoparticles (NPs) are the most widely researched and applied. The definition of nanomaterials recommended by the European Commission (EC) in 2011 (2011/696/EU) is as follows:

A natural, incidental or manufactured material containing particles, in an unbound state or as an aggregate or as an agglomerate and where, for 50% or more of the particles in the number size distribution, one or more external dimensions is in the size range 1 nm–100 nm.

The generally accepted definition of the size of NPs is 1–100 nm (The Royal Society and Royal Academy of Engineering, 2004). However, sometimes particles up to 1000 nm in size are included in nanotechnology. The nanometre size confers some unique properties to NPs, namely large specific surface area (surface area per unit mass), high surface energy and quantum confinement [1]. This improves their biochemical reactivity. These properties are responsible for the unique behaviour and environmental effects of NPs compared with larger particles of the same kind. Nanotechnology is identified by the communication of the EC as a key enabling technology with a global market evaluated at around 11 million tonnes and direct employment of 300,000 to 400,000 jobs (these figures take into account commonly known nanomaterials, i.e. those known to have a particle size between 1 and 100 nm) [2].

The global nanotechnology market is projected to exceed US $125 billion by 2024 of which about 85% consists of NPs [3]. The use of NPs in agriculture, industry, biomedicine and domestic goods is increasing exponentially, and with it, the rate of their release into the environment. The nanomaterials commonly in use are silver (Ag), gold (Au) and nano‐oxides of copper (CuO/Cu_2_O), iron (Fe_3_O_4_/Fe_2_O_3_), cerium (CeO_2_), titanium (TiO_2_), zinc (ZnO), silicon (SiO_2_) and magnesium (MgO) [4]. Of these, NPs of Ag, CuO, TiO_2_, ZnO and SiO_2_ are employed in agriculture as pesticides, fungicides, herbicides or fertilizers [5, 6, 7]. In 2010, it was reported that 63%–91% of the 260,000–309,000 metric tons of worldwide products containing NPs were disposed of in landfills while 8%–28% of them entered the soil [8]. Plants, due to their stationary nature, are in constant interaction with NPs present in the environment which includes soil, water and air. NPs, at sub‐toxic concentrations, are known to act as abiotic stress agents to plants while at higher concentrations they act as phytotoxic agents [9]. NPs, in general, are reported to exert their toxicological effect on plants by the generation of reactive oxygen species (ROS) [10, 11]. It is known that plants respond to oxidative stress by activating both enzymatic and non‐enzymatic anti‐oxidant defence mechanisms to scavenge excess ROS [12]. Correspondingly, NP‐mediated stress also activates the plant's anti‐oxidant machinery.

It is known that plants respond to various biotic and abiotic stress conditions by increased or *de novo* biosynthesis of secondary metabolites [13, 14]. It has been suggested that nanoparticle‐generated ROS may act as a trigger for the induction of secondary metabolism in plants [11]. Secondary metabolites play a defensive role in plants against abiotic as well as biotic stress including pathogens, pests, herbivores and predators. They may act as phytoalexins/phytoanticipins offering protection against pathogen attacks [15, 16, 17], or can resist abiotic stress as physical or chemical protectors or anti‐oxidants of ROS [13]. They may also act as chemical signals in symbiotic interactions with beneficial organisms, and as allelopathic agents to protect plants from rhizosphere competitors [18]. In addition, they also serve as physical and chemical barriers to abiotic stressors and as anti‐oxidants to scavenge ROS [13, 19]. Hence, it can be inferred that stimulation of secondary metabolism by nanoparticle‐mediated ROS will lead to protection of plants from abiotic and biotic stress. Chandra et al. [20], demonstrated in *Camellia chinensis* (tea) *ex vivo* that chitosan NPs (90 ± 5 nm in diameter) could act as an effective elicitor of innate immune response in plants, which upregulated the genes and increased the activity of defence enzymes peroxidase, polyphenol oxidase (PPO), phenylalanine ammonia lyase (PAL), *β*‐1,3‐glucanase as well as anti‐oxidant enzymes superoxide dismutase (SOD) and catalase (CAT). It coincided with an increase in phenolics, particularly flavonoids which play a key role in the defence response. They suggested a role of nitric oxide as a signal molecule in the innate immune response [20].

Many secondary metabolites are useful to mankind as pharmaceuticals, flavouring agents, food additives and industrially important chemicals in textiles, cosmetics etc. [13, 14] The present review focuses on exploring the role of NPs in enhancing the economic value of plants by positively influencing their secondary metabolism, thereby converting a stress response to an economic benefit for mankind. It may be mentioned that the flavour and anti‐oxidant properties of tea, which confer its health benefits, are largely determined by its phenol and polyphenol content [21, 22] and is expected to be enhanced by the treatment with chitosan NPs. [20] Although secondary metabolites play a key role in protecting plants against biotic and abiotic stress, particularly pathogen attacks, which indirectly is an economic benefit, the phytoprotective aspect is beyond the scope of this review, which focuses on the production of secondary metabolites useful to mankind.

The uptake, accumulation and build‐up of NPs in plants vary, and these factors largely depend on the size, shape and the composition of the NPs as well as on the type, size and nature of the plants. The uptake of NPs by plants may occur either *in* *vivo* or *in vitro*, under culture conditions. The *in vivo* routes of uptake include (a) by foliar sprays and (b) through roots from the soil or hydroponic nutrient medium. The *in vitro* routes of administration are through artificial nutrient media in different types of tissue cultures. In case of foliar sprays, NPs primarily enter through stomata into the sub‐stomatal chamber and subsequently into the mesophyll cells. In case of roots, the entry may be through root hairs or epidermal cells into cortical cells. Several mechanisms have been proposed for the entry of NPs into plant cells. Some pathways suggested include entry by being bound to a carrier protein, through aquaporin, ion channels or endocytosis through existing pores or by the creation of new pores [23, 24]. The penetrative capacity by creating new pores is particularly an attribute of carbon nanotubes [25]. Due to high surface area‐to‐mass ratio of NPs compared with the bulk metals they have higher reactivities compared with the surroundings [26]. Consequently, they may readily form complexes with membrane transporters or root exudates before being transported into the plants. Metal‐based NPs may be taken up through the corresponding ionic transporters, since most that have been reported as being taken up by plants include elements for which ion transporters have been identified [27]. After entry into the plant cells, the NP may be transported either apoplastically or symplastically from one cell to another via plasmodesmata [28] and translocated within the plant via the liquid column of xylem or phloem.

## 2 NANOPARTICLES AS ELICITORS OF SECONDARY METABOLITES IN PLANTS *IN VIVO*

**FIGURE 1:**
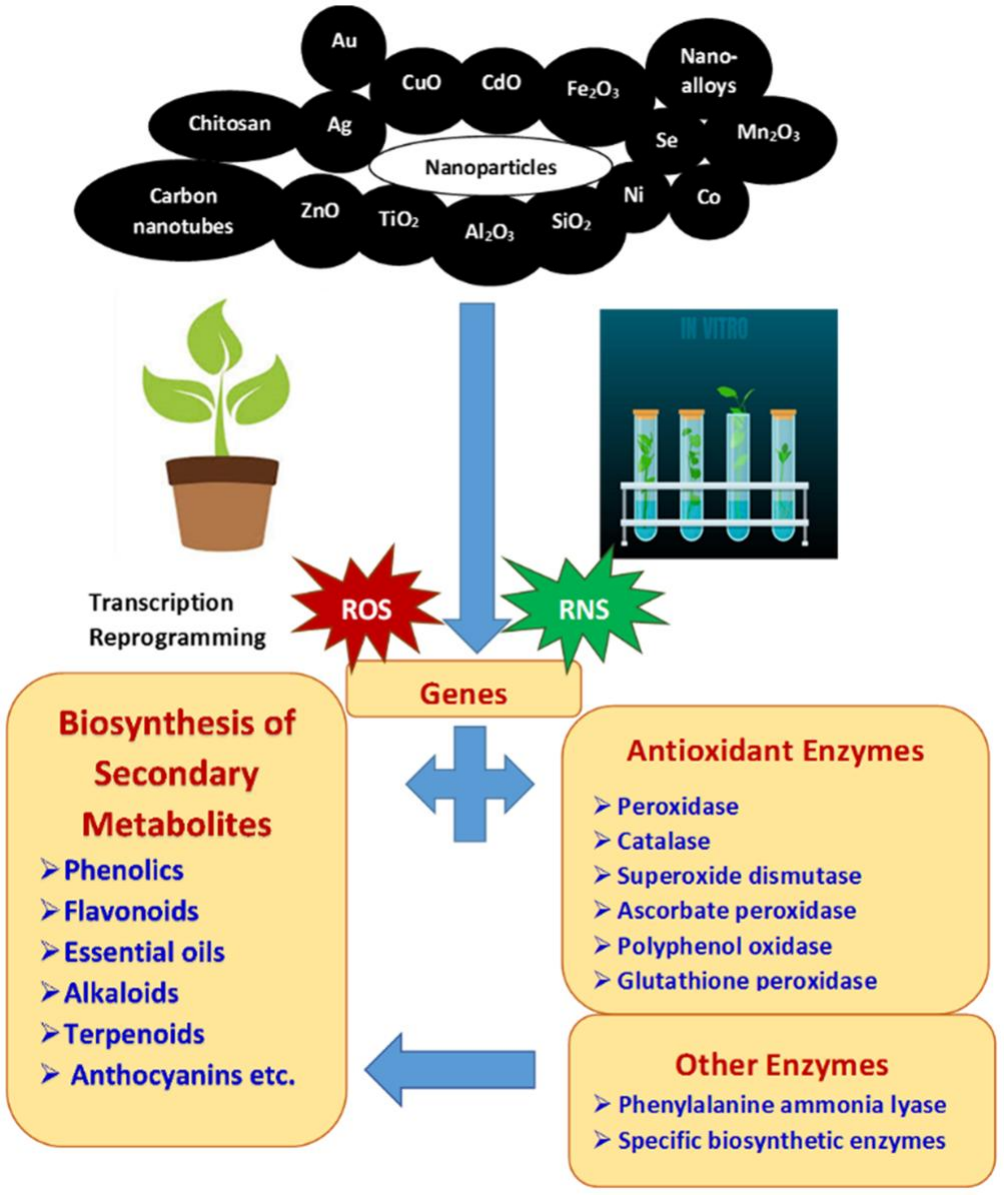
Schematic representation of the elicitation of secondary metabolites by currently known nanoparticles in plants

Many studies have reported the application of nanoparticles to positively modulate the content and/or composition of secondary metabolites in plants *in vivo*. The enhancement of secondary metabolite content is generally reported to have a positive correlation with the activities of enzymes related to oxidative defence and secondary metabolism, and often with the transcription of their corresponding genes (Figure 1 and Table 1).

**TABLE 1:** Elicitation of secondary metabolites by nanoparticles in plants *in vivo*

NP	Size of NPs (nm)	Effective concentration of NPs	Plant species	Mode of application	Effect on secondary metabolism and antioxidant capacity	Effect on ROS, antioxidant and other enzyme activities	Effect on antioxidant and secondary metabolic genes	Reference
A. Metal‐based								
Ag	2–50	10 ppm	*Bacopa monnieri*	Hydroponic	Increase in total phenolics	Increased CAT, POD	_	29
Ag	∼32	60–100 ppm	*Thymus kotschyanus (Thyme)*		Increase in the major compounds of essential oils such as α‐terpinyl acetate at 60 ppm; thymol content was more than twice of carvacrol at all employed concentrations except 100 ppm	_	_	30
Ag	∼35	0.6 mM	*Borago officinalis (borage)*	Foliar	Increase the phenol, tannin and alkaloid contents	_	_	31
Ag	30–50	0.4 mM	*Calendula officinalis (marigold)*	Hydroponic	117% increase in saponin content but decrease in anthocyanin and flavonoid contents	_	_	32
Ag	30–50	1 mM	*Achillea millefolium*	Hydroponic	∼230% increase in essential oil content; increase in flavonoids as well as antibacterial isoprenoids, namely camphor, allo‐ocimene, germacrene, *trans*‐caryophyllene and farnesol; decrease in anthocyanin.	Increase in lipid peroxidation but decrease in radical scavenging activity	_	33
Ag	5–20	40–80 mg L^−1^	*Pelargonium graveolens (geranium)*	Foliar	Increased essential oil content; among the 26 components of the essential oil, the maximum citronellol and geraniol content was found at 40 mg L^−1^, while linalool and citronellyl butyrate content decreased steadily with increasing AgNP levels until 40 mg L^−1^ and increased rapidly thereafter; maximum citronellol/geraniol ratio was obtained at 80 mg L^−1^	_	_	34
Ag	1–20	5–10 mg L^−1^	*Brassica rapa ssp. rapa* (turnip)	Foliar	Increase in secondary metabolism, e.g. anthocyanin	Increase in ROS: H_2_O_2_ and MDA	Induction of secondary metabolism genes, e.g. glucosinolates, anthocyanin	35
Ag		250–500 mg L^−1^	*Brassica rapa ssp. pekinensis* (Chinese cabbage)	Foliar	Increase in secondary metabolism, namely anthocyanin	Increase in ROS: H_2_O_2_ and MDA	Induction of genes of glucosinolates and anthocyanin	36
Ag	8–21	0.2 μg ml^−1^	*Trigonella foenum‐graecum* (fenugreek)	Soil	Enhanced diosgenin synthesis	_	_	37
Ag	∼25	40 mM	*Stevia rebaudiana*	Foliar	Enhancement of glycosides stevioside and rebaudioside A	_	_	38
Ag	∼21.64	30 ppm	*Citrus reticulata* (Kinnow Mandarin)	Foliar	Enhancement of total phenolics and flavonoids; enhancement of antioxidant capacity	_	_	39
Ag		4 or 40 mg/plant	*Cucumis sativus*	Foliar	Increase in phenolics. Enhancement of antioxidant capacity	_	_	40
Ag		200 ppm	*Rosmarinus officinalis* (Rosemary)	Foliar	Enhanced carnosic acid content (>11%) along with that of total flavonoids	_	_	41
CuO	15–32	30 μg ml^−1^	*Citrus reticulata* (Kinnow Mandarin)	Media	Enhancement of total phenolic and flavonoid contents as well as antioxidant capacity	_	_	42
ZnO	8–32							
Cu		1.0 g L^−1^	*Mentha piperita* (peppermint)	Foliar	Increase in essential oil percentage by 20%. Increase in menthol (15%), menthone (25%) and menthofuran (65%) content in the essential oil	_	_	43
CuO		1 ppm	*Withania somnifera* (Ashwagandha)		Enhancement of polyphenol, flavonoid and tannin content along with antioxidant capacity	_	_	44
CuO		1 ppm	*Chicorium intybus* (chicory)		Enhancement of polyphenol, flavonoid and tannin content along with antioxidant capacity	_	_	45
Cu(OH)_2_	∼40	10–20 mg L^−1^	*C* *ucumis* *sativus* (cucumber)	Hydroponic	Increase in ascorbic acid, phenolics as well as amino acids while decreasing citric acid	_	_	46
Au	40	3 μg ml^−1^	*Artemisia absinthum*	Medium	Enhanced total phenolic content (TPC), total flavonoid content (TFC) and antioxidant activity. While TPC and TFC were enhanced more by AgNP treatment, AuNPs showed greater enhancement of radical scavenging capacity	Increased SOD activity and total protein content	_	47
Ag	34							
Cu	26							
CuO	50	250 mg L^−1^	*Solanum lycopersicum* (tomato)	Foliar	Increased vitamin C, lycopene, total phenols and flavonoids in the fruits and enhanced antioxidant capacity.	Increase in antioxidant enzymes CAT and SOD.	_	48,49
					Under salt stress, enriched phenols (16%) in the leaves and the content of vitamin C (80%), glutathione (GSH) (81%) and phenols (7.8%) in the fruit	Under salt stress increased activities of PAL (104%), APX (140%), GPX (26%) SOD (8%) and CAT (93%) in the leaf tissue		
Cu_2_O/Cu	2–20	20–40 mg L^−1^	*Bacopa monnieri*	Hydroponic	Hormetic increase in the contents of saponins, alkaloids, flavonoids as well as antioxidant capacity from 5 mg L^−1^ to a maximum at 40 mg L^−1^, and of phenolics at 20 mg L^−1^, decreasing thereafter	Increase in ROS markers H2O2, MDA; hormetic effect on activities of PAL, SOD, CAT and APX	_	51
Cu	∼50	50 mg L^−1^ +	*Solanum lycopersicum* (tomato)	Foliar	Increase in vitamin C, glutathione, phenol and flavonoid content in fruits, along with decrease in the severity of early blight disease caused by the fungus *Alternaria solani*	Induction of the activity of SOD, APX, GPX and PAL in the leaves, and GPX in the fruit	_	52
Se	2–20	20 mg L^−1^ jointly						
CuO	<50	1 and 10 μM	*Glycyrrhiza glabra* (licorice)	Medium	Enhancement of glycyrrhizin, total phenolic compounds, flavonoids, anthocyanins and proline content	_	_	53
ZnO		1 and 10 μM						
CuO	25–55	100, 250, 500 mg L^−1^ separately	*Solanum melongena (eggplant)*	Sterile filter paper	Enhancement of anthocyanin, flavonoids and phenolics in a concentration‐dependent manner, the effect of NiO NPs being the most pronounced	_	_	54
NiO	10–20							
ZnO	18							
* NP*	*Size of NPs (nm)*	*Effective concentration of NPs*	*Plant species*	*Mode of application*	*Effect on secondary metabolism and antioxidant capacity*	*Effect on ROS, antioxidant and other enzyme activities*	*Effect on antioxidant and secondary metabolic genes*	*Reference*
Fe‐O + *γ* radiation	20	30 ppb + 20 GY	*Lepidum sativum* (cress)	Foliar spray	Increase in contents secondary metabolites like essential oils, phenolics and flavonoids	_	_	55
CdO	7–60	2.03 ± 0.45 × 10^5^ particles cm^−3^	*Hordeum vulgare (barley)*	Foliar	Enhanced ferulic acid and isovitexin content	_	_	56
TiO_2_	10–15	100–200 mg L^−1^	*Salvia officinalis (sage)*	Foliar	Enhanced secondary metabolites such as phenolics, flavonoids and essential oils. Among the major constituents of essential oils, the maximum increase of cis‐Thujene (34.5 %) and 1,8‐cineol (21.2 %) were achieved in plants exposed to 200 mg L^−1^ TiO_2_ NPs, while the maximal content of camphene (12.1 %) was obtained from plants exposed to 1000 mg L^−1^ nano‐TiO_2_ treatment	_	_	57
TiO_2_	10–25	10 ppm	*Dracocephalum moldavica (Moldavian* dragonhead)	Foliar spray	Under normal irrigation increased plant shoot dry mass and essential oils content; under drought stress, increased content of essential oils and some valuable phenolics like rosmarinic acid and chlorogenic acid	Under water‐deficit condition, decreased H2O2 and MDA content, indicating amelioration of water deficit stress	_	58,59
TiO_2_		100, 250 and 500 mg L^−1^	*Oryza sativa L*.(rice)	Hydroponic	Enhanced secondary metabolite as well as amino acid and fatty acid content	_	_	60
TiO_2_	<21	100 mg L^−1^ and 150 mg L^−1^	*Mentha piperita* (peppermint) L.	Foliar spray	Enhanced essential oil content by 39.4% and 105.1%, respectively, over control, simultaneously increasing content and yield of menthol in the essential oil by 9.6% and 124.1%	_	_	61
TiO_2_	∼14	90 mg L^−1^	*Vetiveria zizanioides L*. (vetiver grass/khus)	Foliar	Increased the content and yield of essential oil by 23.6% and 55.1%, respectively. The content and yield of khusimol, the main ingredient of the essential oil was found to be enhanced by 24.5% and 93.2%, respectively	_	_	62
TiO_2_		50 and 100 mg L^−1^	*Nigella sativa L. (black cumin)*	Foliar spray	Enhanced thymoquinone synthesis	_	Stimulated the expression of the *Geranyl diphosphate synthase (GPPS)*, the key gene in thymoquinone synthesis. The effect of TiO_2_ NP > SiO_2_ NP	63
SiO_2_								
TiO_2_	∼25	25 mM	*Tanacetum parthenium L*. (feverfew)	Soil	Enhanced parthenolide synthesis	_	SiO_2_ NP: Upregulated the expression of the genes related to parthenolide synthesis, *TpCarS*, *COST* and *TpGAS*. TIO_2_ NPs: Upregulated *COST* and *TpGAS*, downregulated *TpCarS*	64
SiO_2_	10–15							
ZnO	12–24	100 ppm	*Capsicum annum L*.	Synthetic matrix	Accumulation of phenolics, flavonoids and tannins while enhancing the anti‐oxidant capacity of the seedlings while enhancing seed germination	_	_	65
Zn	30–70	5, 15 and 25 mg L^−1^	*Brassica napus L*. (turnip)	Foliar	Increased total flavonoid content while decreasing total phenolic content	Increased SOD and antioxidant enzymes		66
ZnO	∼5–12	60 ppm	*Raphanus sativus cv. Champion* (red radish)	Foliar with chicken manure in soil	Enhanced the concentration of anthocyanins, phenols, tannins, flavonoids as well as crude protein and carbohydrates contents in roots			67
FeO	∼2–6	50 ppm						
Bimetallic nano‐alloys of Au, Ag and Cu	18–48	30 μg ml^−1^	*Eruca sativa*	Medium	Enhanced phenolics and flavonoids along with anti‐oxidant capacity. The effect of Cu in the NPs was more pronounced than that of Au and Ag. Smaller NPs caused more toxic stress	_	_	68
Zn‐Ag	25–40	20 mg L^−1^	*W. somnifera L. Dunal* (Ashwagandha)	Foliar	Enhanced withanolide synthesis	Both effects correlated well with activity of antioxidant enzymes as well as rates of transpiration, photosynthesis, Calvin cycle and carbohydrate metabolism		69
Nanoalloy (19:1,13:1)					Negative effect on withanolide synthesis			
Zn‐Ag NPs (9:1, 1:1), Cd‐Se Quantum dots, Ni NPs								
b. Non‐metal oxide NPs								
SiO_2_		50 and 100 mg L^−1^	*Mentha piperita L*. (peppermint)	Foliar spray	Increased essential oil content while enriching the menthol content but decreasing menthone and menthyl acetate in the essential oil	_	_	70
c. Carbon‐based NPs								
Chitosan	90 ± 5	0.001% (w/v)	*Camellia chinensis* (tea)	Foliar *ex vivo*	Increase in phenolics, particularly flavonoids	Increased activity of defence enzymes POD, PPO, PAL, *β*‐1,3‐glucanase as well as antioxidant enzymes SOD and CAT	Upregulation of the genes of PPO, *β*‐1,3‐glucanase, PAL, thaumatin‐like protein (TLP), SOD, CAT as well as flavonoid biosynthetic genes cinnamate 4‐hydroxylase (C4H), flavonoid 3‐hydroxylase (F3H) and anthocyanidin reductase (ANR)	20

Abbreviations: APX, ascorbate peroxidase; CAT, catalase; GPX, glutathione peroxidase; NP, nanoparticle; PAL, phenylalanine ammonia lyase; POD, peroxidase; PPO, polyphenol oxidase; ROS, reactive oxygen species; SOD, superoxide dismutase.

### 2.1 Metal, metal oxide and metal alloy nanoparticles

*Silver nanoparticles* (AgNPs) have been the most extensively used as elicitors of secondary metabolism in plants in laboratory experiments, although their high cost is likely to be a limiting factor in their commercial application. In one of the earliest reports, AgNPs (diameter 2–50 nm) synthesized using aqueous leaf extract of *Acalypha indica* L. were found to enhance total phenolic content (TPC) along with catalase and peroxidase activity in hydroponically treated *Bacopa monnieri* (Linn.) Wettst. plants. However, the stress response was milder in comparison to Ag^+^ ions released from AgNO_3_ solution [29]. Aghajani et al. [30], reported the effect of AgNPs (diameter ∼32 nm) exposure (3 h at 20, 40, 60, 80 and 100 ppm) on essential oil content and composition in *Thymus kotschyanus* Boiss. & Hohen. There was an increase in the major compounds of essential oils such as *α*‐terpinyl acetate at 60 ppm exposure and the thymol content was more than twice of carvacrol at all employed concentrations of AgNPs except 100 ppm. However, the minor components of the essential oil were not significantly altered under the conditions of the experiment. In *Borago officinalis* L. (borage) foliar application of AgNPs (diameter ∼35 nm) was found to increase the phenol, tannin and alkaloid contents along with other vegetative and phytochemical properties, the most effective concentration being 0.6 mM [31]. Hydroponically grown *Calendula officinalis* L. (marigold) plants, when treated with 0.4 mM AgNPs (diameter 30–50 nm) and 100 mM methyl jasmonate (MeJa) showed 117% increase in saponin content but a decrease in anthocyanin and flavonoid contents [32]. Similar effects were observed in the medicinal herb *Achillea millefolium* L., which on elicitation with AgNPs (diameter 30–50 nm) and MeJa showed an increase of approximately 230% in essential oil content. There was an increase in flavonoids as well as some precious medicinal compounds such as anti‐bacterial isoprenoids, namely camphor, allo‐ocimene, germacrene, *trans*‐caryophyllene and farnesol which possess anti‐bacterial, anti‐fungal, anti‐inflammatory and anti‐cancer properties. There was an increase in lipid peroxidation but a decrease in anthocyanin content and radical scavenging activity [33]. In *Pelargonium graveolens* (geranium) foliar application of AgNPs (size 5–20 nm) enhanced essential oil yield, the maximum being at 40 mg L^−1^ concentration. Among the 26 components of the essential oil, the maximum citronellol and geraniol content was found at 40 mg L^−1^, while linalool and citronellyl butyrate content decreased steadily with increasing AgNP levels until 40 mg L^−1^ and increased rapidly thereafter. Maximum citronellol/geraniol ratio was obtained at 80 mg L^−1^ [34]. AgNPs (1–20 nm; 5 and 10 mg L^−1^) were demonstrated to induce most of the genes related to secondary metabolism (glucosinolates, anthocyanin) in *Brassica rapa* ssp. *rapa* L. (turnip) seedlings along with an increase in the content of anthocyanin and malondialdehyde as well as hydrogen peroxide, indicating oxidative stress [35]. Similar gene induction and anti‐oxidant activities were observed with AgNPs at higher concentrations (>250 mg L^−1^) in *Brassica rapa* ssp. *pekinensis* (Chinese cabbage) seedlings [36]. Treatment of *Trigonella foenum‐graecum* L. (fenugreek) seedlings with AgNPs (diameter 8–21 nm) were reported to significantly enhance plant growth as well as diosgenin biosynthesis [37]. AgNPs (diameter ∼25 nm) were reported to act as positive elicitors of the glycosides stevioside and rebaudioside A in *Stevia rebaudiana* (B), after spray treatment, the maximum enhancement occurring at a concentration of 40 mM [38]. In *Citrus reticulata* (Kinnow Mandarin) AgNPs (size ∼21.64 nm) synthesized using leaf extracts of *Moringa oleifera*, on exogenous application*,* enhanced the synthesis of total flavonoids and phenolics at a concentration of 30 ppm thereby increasing its anti‐oxidant capacity and offering resistant against brown spot disease caused by *Alternaria alternate* [39]. In *Cucumis sativus* (cucumber) AgNPs (4/40 mg/plant) were reported to activate oxidative defence response by an increase of phenolics [40]. In hydroponically grown *Rosmarinus officialis* L. (Rosemary) foliar application AgNPs at 200 ppm for 12 days was found to enhance carnosic acid content by more than 11% along with that of total flavonoids [41].

*Copper or copper oxide nanoparticles* (Cu/CuO NPs) have also been reported to be effective elicitors of secondary metabolism in plants. Plantlets of *Citrus reticulata*, when germinated *in vitro* in media supplemented separately with CuO NPs (15–32 nm) and ZnO NPs (8–32 nm) (green synthesized using white leaves of *Allium cepa* L.) at concentrations of 30 μg ml^−1^, showed significant enhancement of total phenolic and flavonoid contents as well as anti‐oxidant capacity [42]. Foliar treatment of *Mentha piperata* L. (peppermint) plants with CuNPs (1.0 g L^−1^) was reported to increase chlorophyll content and essential oil percentage by 35% and 20%, respectively. The menthol, menthone and menthofuran content in the essential oil were up to 15%, 25% and 65% higher than in control, respectively [43]. CuO NPs were found to significantly enhance polyphenol, flavonoid and tannin content along with anti‐oxidant capacity in roots of the Indian medicinal plants *Withania somnifera* L. Dunal (Ashwagandha) [44] as well as in *Chicorium intybus* L. (chicory) [45]. Hydroponic application of Cu(OH)_2_ nanopesticides (primary diameter ∼40 nm, hydrodynamic diameter 2590 ± 1138 nm in deionized water) at concentrations of 10 and 20 mg L^−1^ in *Cucumis sativus* L. (cucumber) was found to increase ascorbic acid, phenolics as well as amino acids while decreasing citric acid [46]. *In vitro* germinated seedlings of *Artemisia absinthium* treated with NPs of Au (40 nm), Ag (34 nm) and Cu (26 nm) showed enhanced TPC, total flavonoid content (TFC), anti‐oxidant activity, SOD activity and total protein content. While TPC and TFC were enhanced more by AgNP treatment, gold nanoparticles (AuNPs) showed greater enhancement of radical scavenging capacity [47]. Foliar application of CuO NPs (50 nm) in plants of *Solanum lycopersicum* L. (tomato) enhanced fruit quality by stimulating greater accumulation of bioactive compounds such as vitamin C, lycopene, total phenols and flavonoids in the fruits and enhancing anti‐oxidant capacity along with increasing anti‐oxidant enzymes CAT and SOD. The best results were obtained with a CuNP concentration of 250 mg L^−1^ [48]. Under salt stress the same NP at 250 mg L^−1^ enhanced the Cu concentration in the tissues of tomato while enriching phenols (16%) in the leaves and the content of vitamin C (80%), glutathione (GSH) (81%) and phenols (7.8%) in the fruit compared with the control. This was accompanied by an increase in the activities of anti‐oxidant enzymes phenylalanine ammonia lyase (PAL), ascorbate peroxidase (APX), glutathione peroxidase (GPX), SOD and catalase (CAT) in the leaf tissue by 104%, 140%, 26%, 8% and 93%, respectively [49]. While the others are anti‐oxidant defence enzymes, PAL is the first enzyme of the general phenylpropanoid pathway that catalyses the deamination of phenylalanine to cinnamic acid and plays a key role in diverting aromatic amino acids from primary metabolism to the phenylpropanoid pathway of secondary metabolism [50]. All of these afforded better salt stress tolerance and enhanced anti‐oxidant defence to the plant [49]. In studies with Cu_2_O/Cu NPs (2–20 nm) in hydroponically grown *Bacopa monnieri* (L.) Pennell Plants *in vivo* it was observed that the contents of saponins, alkaloids, flavonoids as well as anti‐oxidant capacity was observed to increase from 5 mg L^−1^ to a maximum at 40 mg L^−1^, and of phenolics at 20 mg L^−1^, decreasing thereafter, an effect known as hormesis. A concentration of 100 mg L^−1^ was detrimental to the production of secondary metabolites, presumably due to metabolic toxicity which inactivates the enzymes. A similar trend was observed in the activities of PAL and anti‐oxidant enzymes SOD, CAT and APX, while there was consistent increase in ROS marker H_2_O_2_ and MDA [51]. Foliar application of CuNPs (∼50 nm) and Selenium NPs (2–20 nm) jointly in *Solanum lycopersicum* (tomato) decreased the severity of early blight disease caused by the fungus *Alternaria solani*, while simultaneously increasing vitamin C, glutathione, phenol and flavonoid content in fruits, thereby improving fruit quality. The effect was correlated with the induction of the activity of the enzymes SOD, APX, GPX and PAL in the leaves, and the enzyme GPX in the fruit [52]. Enhancement of glycyrrhizin, total phenolic compounds, flavonoids, anthocyanins and proline content was reported in seedlings of *Glycyrrhiza glabra* (licorice) seedlings after elicitation by nano‐oxides of Cu and Zn (size <50 nm). [53]. *In vitro* grown seedlings of *Solanum melongena* L. (eggplant) treated with nano‐oxides of Cu (25–55 nm), Ni (10–20 nm) and Zn (18 nm) showed enhancement of secondary metabolites anthocyanin, flavonoids and phenolics in a concentration‐dependent manner, the effect of NiO NPs being the most pronounced [54].

*Iron oxide* (Fe‐O) NPs (20 nm), when applied synergistically with Gamma irradiation, were reported to result in a significant increase in contents secondary metabolites like essential oils, phenolics and flavonoids in *Lepidum sativum* L. (cress) cultivated in sandy soil with a low quantity of saline water [55].

*Cadmium oxide* (CdO) NPs (size 7–60 nm; concentration 2.03 ± 0.45 × 10^5^ particles cm^−3^) enhanced ferulic acid and isovitexin content in *Hordeum vulgare* L. (barley) plants [56].

*Titanium dioxide* (TiO_2_) NPs (10–15 nm), on application in *Salvia officinalis* L. (sage) plants, enhanced secondary metabolites such as phenolics, flavonoids and essential oils, the maximum increase being at concentrations of 100 and 200 mg L^−1^. Among the major constituents of essential oils, namely monoterpenes, including Camphene, *p*‐Cymene, 1,8‐Cineol, cis‐Thujene and Camphor, the maximum increase of cis‐Thujene (34.5%) and 1,8‐Cineol (21.2%) were achieved in plants exposed to 200 mg L^−1^ TiO_2_ NPs, while the maximal content of Camphene (12.1%) was obtained from plants exposed to 1000 mg L^−1^ nano‐TiO_2_ treatment [57]. On foliar application in *Dracocephalum moldavica* L. (Moldavian Dragonhead) plants under normal irrigation, TiO_2_ NP (10–25 nm) at 10 ppm concentration increased plant shoot dry mass and essential oils content. Under water‐deficit condition, plants treated with 10 ppm TiO_2_ NPs had higher proline and significantly lower H_2_O_2_ and malondialdehyde content as compared with untreated plants, indicating amelioration of water deficit stress [58]. Under drought stress, TiO_2_ NPs increased the content of essential oils and some valuable phenolics like rosmarinic acid (RA) and chlorogenic acid in the same plant [59]. TiO_2_ NPs have been demonstrated to enhance secondary metabolite as well as amino acid and fatty acid content correlated with crop quality in *Oryza sativa* L. (rice) [60]. In *Mentha piperita* L. (peppermint), treatment with TiO_2_ NPs (<21 nm) at concentrations of 100 and 150 mg L^−1^ was reported to significantly enhance essential oil content by 39.4% and 105.1%, respectively, over control, simultaneously increasing content and yield of menthol in the essential oil by 9.6% and 124.1% [61]. On foliar application at a concentration of 90 mg L^−1^ in *Vetiveria zizanioides* L. Nash (vetiver grass/khus), TiO_2_ NPs (size ∼14 nm), increased the content and yield of essential oil by 23.6% and 55.1%, respectively. The content and yield of khusimol, the main ingredient of the essential oil was found to be enhanced by 24.5% and 93.2%, respectively. This coincided with an enhancement in chlorophyll content and photochemical efficiency of PSII [62]. In *Nigella sativa* L. (Black cumin), treatment of plants in early flowering stage with SiO_2_ and TiO_2_ NPs was found to stimulate the expression of the *Geranyl diphosphate synthase* (*GPPS*) gene, which is the key gene in the synthesis of the secondary metabolite thymoquinone, in a concentration‐dependent manner. The effect of TiO_2_ NPs was more pronounced than that of SiO_2_ NPs [63]. Treatment of *Tanacetum parthenium* L. (feverfew) plants separately with TiO_2_ (∼25 nm) and SiO_2_ NPs (10–15 nm), at concentrations of 25 mM augmented parthenolide synthesis. The expression of the genes related to parthenolide synthesis, *TpCarS*, COST and *Tp*GAS were all upregulated by SiO_2_ NPs, and TiO_2_ NPs upregulated COST and *Tp*GAS while downregulating *TpCarS* [64].

*Zinc oxide (ZnO)* NPs (12–24 nm), when applied to *Capsicum annum* L. seeds at concentrations of 100 ppm and higher before germination, were found to inhibit seedling radical growth and promote the accumulation of phenolics, flavonoids and tannins while enhancing the anti‐oxidant capacity of the seedlings [65]. Biogenically synthesized Zn NPs (size 30–70 nm) using leaves of *Mentha arvensis* L. significantly increased TFC and SOD in *Brassica napus* L. (turnip) while decreasing TPC [66]. The combined foliar application of ZnO NPs (∼5–12 nm) synthesized using *Leuconostoc mesenteroides* (lactic acid bacteria) and FeO NPs (∼2–6 nm) synthesized using *Saccharomyces cerevisiae* (yeast) along with chicken manure was reported to significantly enhance the concentration of anthocyanins, phenols, tannins, flavonoids as well as crude protein and carbohydrates contents in roots of field‐grown *Raphanus sativus* cv. Champion (red radish) compared with a single treatment [67].

*Bimetallic alloy NPs* of Au, Ag and Cu present in different proportions were demonstrated to induce production of secondary metabolites like phenolics and flavonoids in germinating seedlings of the medicinal plant *Eruca sativa* at concentrations of 30 μg ml^−1^, along with enhancement of anti‐oxidant capacity. Smaller NPs induced more toxic stress while the effect of Cu in the NPs was more pronounced than that of Au and Ag [68]. In the ayurvedic medicinal plant *Withania somnifera* L. Dunal (Ashwagandha), alloy Zn–Ag NPs (25–40 nm) in the molar ratio of 19:1 and 3:1 were found to enhance withanolide content while Zn–Ag NPs (9:1, 1:1), Cd–Se Quantum dots and Ni NPs were found to have a negative effect on withanolide biosynthesis and content both *in vivo* and *in vitro*. The effect on withanolide synthesis correlated well with the activity of anti‐oxidant enzymes as well as rates of transpiration, photosynthesis, Calvin cycle and carbohydrate metabolism [69].

### 2.2 Non‐metal oxide nanoparticles

*Silicon dioxide* (SiO_2_) NPs (50 and 100 mg L^−1^), on foliar application, were demonstrated to significantly augment essential oil content in *Mentha piperita* L. (peppermint) while enriching the menthol content but decreasing menthone and menthyl acetate in the essential oil [70].

### 2.3 Carbon‐based nanoparticles

As mentioned earlier, Chandra et al. [20] demonstrated in *Camellia chinensis* (tea) *ex vivo* that *chitosan NPs* (90 ± 5 nm in diameter) caused as increase in phenolics, particularly flavonoids which coincided with upregulation of the genes and increased activity of defence enzymes peroxidase, PPO, PAL, *β*‐1,3‐glucanase as well as anti‐oxidant enzymes SOD and catalase (CAT). Upregulation of the genes of PPO, *β*‐1,3‐glucanase, PAL, thaumatin‐like protein (TLP), anti‐oxidant enzymes SOD, CAT as well as flavonoid biosynthetic genes cinnamate 4‐hydroxylase (C4H), flavonoid‐3‐hydroxylase (F3H) and anthocyanidin reductase (ANR) were also observed.

## 3 NANOPARTICLES AS ELICITORS OF SECONDARY METABOLITES IN PLANTS IN CULTURE

### 3.1 Metal, metal oxide and metal alloy nanoparticles

**FIGURE 2:**
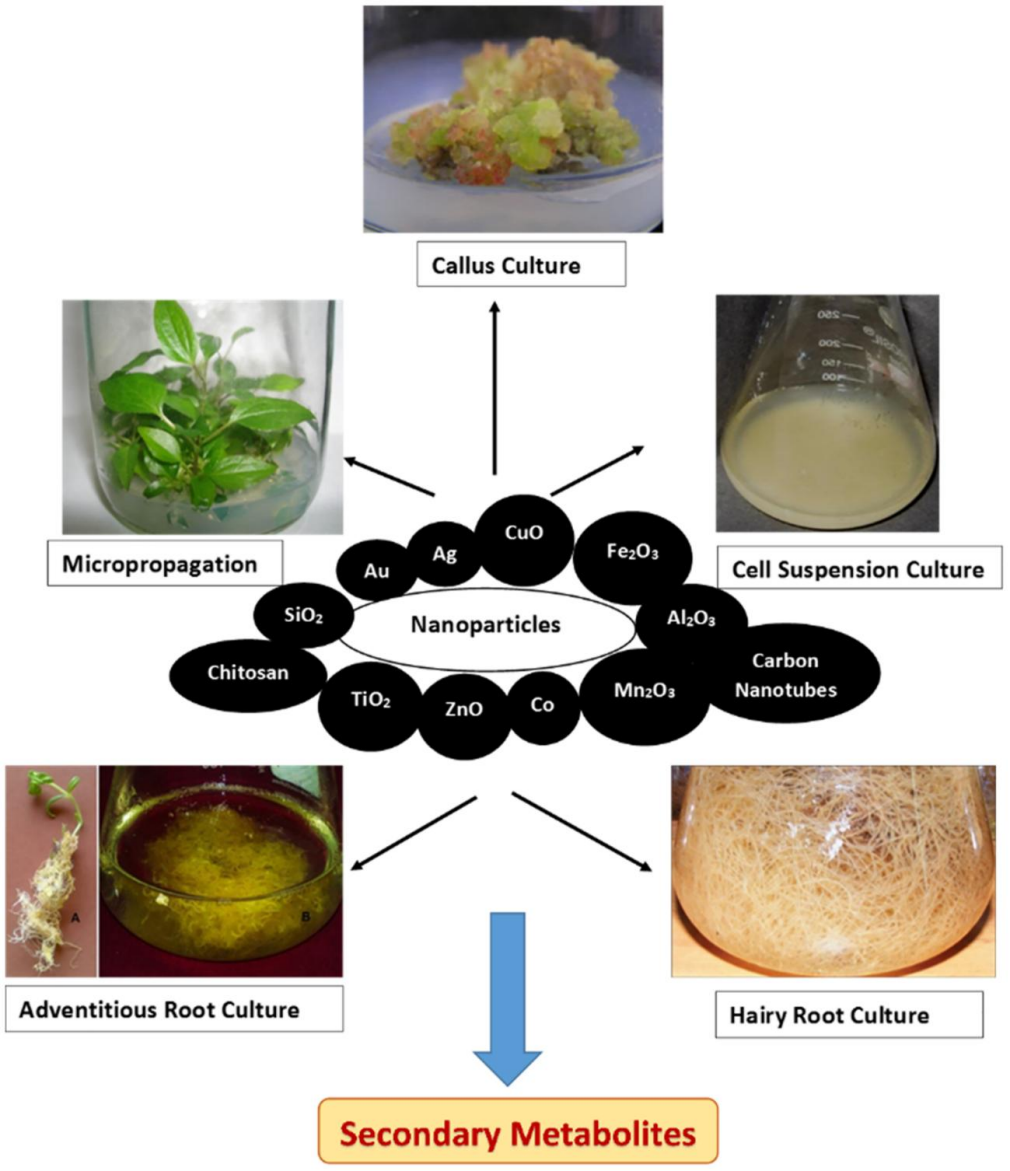
Schematic representation of biosynthesis of secondary metabolites by currently known nanoparticles in plant tissue cultures

*In vitro* cultures of plants supplemented with nanoparticles are emerging as an important technology for the uniform production of high quantities of economically important secondary metabolites (Figure 2). The nanoparticles not only act as elicitors of secondary metabolites but also as a source of micronutrients, and sometimes as anti‐microbial agents and stimulators of callus induction, organogenesis, shoot growth and root initiation [71] (Table 2).

**TABLE 2:** Elicitation of secondary metabolites by nanoparticles in plants in culture

NP	Size of NPs (nm)	Effective concentration of NPs	Plant species	Type of culture	Effect on secondary metabolism and antioxidant capacity	Effect on ROS, antioxidant and other enzyme activities	Effect on antioxidant and secondary metabolic genes	Reference
A. Metal‐based								
Ag		1.2 mg L^−1^	*Calendula officinalis L. (marigold)*	Callus	Enhanced essential oil content with increase in all components *α*‐pynene, *β*‐pynene, *ρ*‐pynene, *α*‐thujene, Calendula glycoside, *α*‐cadinene, cadinol, t‐muurolol, 1,8‐cineol, limonene	Increased lipid peroxidation	_	72
Ag		0.625 mg ml^−1^	*Aloe vera L*.	Suspension	Enhanced aloin content after 48 h of exposure which gradually declined thereafter	_	_	73
TiO_2_		120 mg L^−1^						
Ag			*Capsicum frutescens L*.	Callus	Increase in capsaicin content compared with fruits of C. annuum and C. frutescens	_	_	74
Ag		5 and 10 ppm	*Corylus avellana L. (hazel)*	Cell suspension	Production of anticancer compounds taxanes, taxol and baccatin III along with total soluble phenols, was enhanced about two‐fold at 5 ppm but declined at 10 ppm concentration	_	_	75
Ag	35 ± 15	25 and 50 mg L^−1^	*Vanilla planifolia (vanilla)*	Micropropagation	Hormetic effect on total phenolic content and antioxidant capacity with increase at 25 and 50 mg L^−1^ but decrease at 100 mg L^−1^	_	_	76
Ag	1–20	5 mg L^−1^	*Momordica charantia L. (bitter gourd)*	Cell suspension	Enhanced total phenolic and flavonoid contents; increase in the contents of flavonols hydroxybenzoic and hydroxycinnamic acids	_	_	77
Ag		45 mg L^−1^	*Stevia rebaudiana L*.	Callus	Enhanced stevioside production.	_	_	78
Ag	40	90 μg L^−1^	*Caralluma tuberculata*	Callus	With growth regulators, enhanced production of phenolics (TPC: 3.0 mg), flavonoids (TFC: 1.8 mg) and antioxidant activity (90%), respectively	With growth regulators enhanced activities of PAL (5.8 U/mg); without growth regulators enhanced activities of antioxidant enzymes SOD, POD, CAT and APX.	_	79
Ag		30 μg L^−1^ each alone or in combination in different proportions	*Prunella vulgaris L. (self‐heal)*	Callus	In presence of NAA (2 mg L^−1^) enhanced secondary metabolite production. The Ag‐Au NPs (1:3) induced maximum production of phenolics and flavonoids; Ag‐Au NPs (3:1) without NAA enhanced antioxidant activity (87.85%)	AgAu (1:3) and AuNPs alone enhanced SOD and POD enzymes to the maximum extent	_	80
Au								
Ag	27.5 ± 4.8	10 and 50 mg dm^−3^	*Lavandula angustifolia (lavender)*	Micropropagation	Altered the essential oil composition. There was a decrease in lower molecular weight compounds (e.g. α‐pinene and β‐pinene, camphene, δ‐3‐carene, p‐cymene, 1, 8‐cineole, trans‐pinocarveol and camphoriborneol), which were substituted by higher molecular weight compounds [τ‐cadinol 9‐cedranone and α‐cadinol 9‐cedranone, cadalene, α‐bisabolol, cis‐14‐nor‐muurol‐5‐en‐4‐one, (E,E)‐farnesol]	_	_	81
Au	24.2 ± 2.4							
Cu	∼1–2.7	0.5 mg L^−1^	*Mentha longifolia*	Clonal micropropagation	Increased essential oil content by 2.226% and 2.19%, respectively	_	_	82
Co	∼1.3–3	0.8 mg L^−1^						
CuO	∼47	10 mg L^−1^	*Stevia rebaudiana Bert*.	Micropropagation	Increase in major steviol glycosides (rebaudioside A and stevioside) accompanied by an increase in total phenolic and flavonoid contents as well as antioxidant activity	_	_	83
CuO		50, 100 and 150 mg L^−1^	*Solanum nigrum L*.	Callus	Enhance phenolic content	Enhanced MDA content and activities of antioxidant defence enzymes POD, PPO and of PAL		84
CuO	25–55	3 mg L^−1^	*Gymnema sylvestre*	Cell suspension	Induced a nine‐fold increase in the production of gymnemic acid II and phenolic compounds compared with control	_	_	85
Al_2_O_3_	∼100–500	10–100 μg ml^−1^	*Nicotiana tabacum L.(tobacco)*	BY‐2 cell suspension	Increase in the phenolic content in a dose‐dependent and time‐dependent manner	_	_	86
Co	10	5 mg L^−1^	*Artemisia annua*	Cell suspension	Enhanced artemisinin production (2.25‐fold) after 24 h	_	Downregulation of the expression of two artemisinin biosynthesis genes *SQS* and *DBR2*	87
Mn_2_O_3_		25 mg L^−1^	*Atropa belladonna (deadly nightshade)*	Shoot tip	Enhanced phenolics, flavonoids and alkaloids in a dose‐dependent manner. Enhanced free radical scavenging activity	Activated antioxidant defence enzymes SOD, POD, CAT and APX	_	88
				Micropropagation				
Fe_2_O_3_ + static magnetic field		100 ppm + 30 mT	*Dracocephalum polychaetum*	Cell suspension	Enhanced the contents of total phenolics, flavonoids, anthocyanins and lignin. There was an increase in the content and liberation rate of medicinal compounds such as rosmarinic acid, naringin, apigenin, thymol, carvacrol, quercetin and rutin	Increase in MDA and the activities of the enzymes PPO and PAL	_	89
TiO_2_		4.5 and 6.0 mg L^−1^	*Cicer arietinum (chickpea)*	Callus	Increased the content of gallic acid, chlorogenic acid, o‐coumaric acid, tannic acid and cinnamic acid	_	_	90
Perlite	14.51–23.34	25‐200 mg L^−1^	*Hypericum perforatum (St. John's wort)*	Callus	Increase in the content, variety and number of volatile compounds as well as in the amounts of hypericin and pseudohypercin	_	_	91
TiO_2_‐perlite nanocomposites	15.5–24.61							
ZnO, Fe_2_O_3_		100 ppb	*H. perforatum (St. John's wort)*	Cell suspension	Increased the production of hypericin and hyperforin	_	_	92
ZnO		75 mg L^−1^	*Lilium ledebouri*	Micropropagation	Hormetic effect with maximum content of phenolics and flavonoids concentrations of 75 and 25 mg L^−1^, respectively	_	_	93
		25 mg L^−1^						
ZnO	<100	500–1500 mg L^−1^	*Brassica nigra (Black mustard)*	Callus with organogenesis	Increased phenolic and flavonoid production in a concentration‐dependent manner while enhancing antioxidant and reducing capacity	_	_	94
ZnO	34	1 mg L^−1^	*Stevia rebaudiana*	Shoot micropropagation	Enhanced production of steviol glycosides (rebaudioside A and stevioside) as well as total phenolic and flavonoid contents along with antioxidant capacity, but the effect declined at higher concentration	_	_	95
ZnO	24	1 ppm	*Bacopa monnieri*	Cell suspension	Two‐fold increase in the content of the saponin bacoside A while it was suggested that ZnO NPs possibly have an effect on the isoprenoid pathway of biosynthesisIt was suggested that ZnO NPs possibly have an effect on the isoprenoid pathway of biosynthesis	_	Downregulation of the expression of the *HMG CoA reductase* gene which controls the rate‐limiting step in bacoside A biosynthesis	96
ZnO	<35	100 mg L^−1^	*Linum usitatissimum*	Cell suspension	Repeated elicitation enhanced the production of lignans in 15 days, and of phenolics, flavonoids and neolignans at 25 days	_	_	97
ZnO	34	150 mg L^−1^	*Thymus vulgaris, T. daenensis and T. kotschyanus*	Callus	Enhanced thymol and carvacrol production. The highest increases for thymol and carvacrol were achieved with 150 mg L^−1^ in T. *kotschyanus and T*. daenesis, respectively	_	_	98
Au‐Cu nanoalloy		30 μg L^−1^	*Stevia rebaudiana*	Submerged adventitious root	Stimulated biomass production and enhanced the total content of phenolics and flavonoids as well as antioxidant capacity. Maximum effect was seen with AuCu (1:3) NPs	_	_	99
b. Carbon‐based nanoparticles								
Chitosan	∼200–500	1 mg L^−1^	*Capsicum annuum L*	Micropropagation	Enhanced the contents of soluble phenols, proline and alkaloid while amplifying organogenesis	Enhancement in the activities of the enzymes POD, CAT and PAL	_	100
Multi‐walled carbon nanotubes	Diameter 5–15	25 and 50 μg L^−1^	*Satureja khuzestanica*	Callus	Enhanced the content of phenolics, flavonoids, rosmarinic acid and caffeic acid	Enhanced the activity of oxidative enzymes PPO, POD and PAL	_	101
Multi‐walled carbon nanotubes		50–150 mg L^−1^	*Catharanthus roseus (rose periwinkle)*	*In vitro* seed germination and callus	Enhanced alkaloid and phenol contents 1.7‐fold and 23%, respectively	Enhancement of activities of CAT, POD, and PAL	Upregulation of the deacetylvindoline‐4‐O‐acetyltransferase (*DAT*) gene	102
c. Elicitation in hairy root cultures								
Ag‐SiO_2_ core–shell NPs	101.8 ± 8.9	900 mg L^−1^	*Artemisiaannua*	Hairy root	Enhanced artemisinin production from 1.67 to 2.86 mg g−1 dry weight compared with control	Increase in H_2_O_2_ generation, lipid peroxidation and CAT activity	_	103
Ag	50–60	2 mg ml^−1^	*Datura metel*	Hairy root	Enhanced atropine production by 1.147‐fold, 1.117‐fold, and 2.42‐fold in comparison to the control samples after 12, 24, and 48 h of treatment, respectively	_	_	104
AgNP	1–20		*Brassica rapa ssp. rapa* (turnip)	Hairy root	Elevated levels of glucosinolates (glucoallysin, glucobrassicanapin, sinigrin, progoitrin, gluconapin, 4‐methoxygluco‐brassicin, 4‐hydroxyglucobrassicin, gluco‐brassicin, neoglucobrassicin and gluconasturtin)	Increase in H_2_O_2_ and MDA	Upregulation of the corresponding transcripts *MYB34,* MYB51, *MYB28 and MYB29*	106
					Increase in total phenolic and flavonoid contents and. This was accompanied by enrichment of the phenolic compounds (flavonols, hydroxybenzoic and hydroxycinnamic acids).		Upregulation of their gene expression *(PAL, CHI and FLS)*	
					Increased free radical scavenging activity.			
CuO		100 mg L^−1^	*Brassica rapa ssp. pekinensis* (Chinese cabbage),	Hairy root	Enhanced contents of glucosinolates (gluconasturtin, glucobrassicin, 4‐methoxyglucobrassicin, neoglucobrassicin, 4‐hydroxyglucobrassicin, glucoallysin, glucobrassicanapin, sinigrin, progoitrin and gluconapin)	_	Upregulation of the corresponding transcript *MYB34, MYB122, MYB28* and *MYB29*	107
					Increase in total phenolic and flavonoid contents and. This was accompanied by enrichment of the phenolic compounds (flavonols, hydroxybenzoic and hydroxycinnamic acids)		Upregulation of their gene expression *(PAL, CHI and FLS)*	
					Increased free radical scavenging activity			
SiO_2_		100 mg L^−1^	*Dracocephalum kotschyi*	Hairy root	About 8.26‐fold increase in the content of rosmarinic acid (RA) compared with control, after 48 h exposure time. Anticancer flavonoids including xanthomicrol, cirsimaritin and isokaempferide increased 13‐fold, 13.42‐fold and 10‐fold, respectively, compared with the control	_	Upregulation in the phenylalanine ammonia‐lyase (*pal*) and rosmarinic acid synthase (*ras*) gene expressions	108
Fe‐O		75 mg L^−1^	*D. kotschyi*	Hairy root	9.7‐fold, 11.87‐fold, 3.85‐fold and 2.27‐fold enhancement in the contents of rosmarinic acid, xanthomicrol, cirsimaritin and isokaempferide, respectively, compared with control after 24 h exposure	Enhanced APX, CAT and SOD activities	Upregulation of *pal* and *ras* gene expressions	109

Abbreviations: APX, ascorbate peroxidase; CAT, catalase; GPX, glutathione peroxidase; NP, nanoparticle; PAL, phenylalanine ammonia lyase; POD, peroxidase; PPO, polyphenol oxidase; ROS, reactive oxygen species; SOD, superoxide dismutase.

*Silver nanoparticles* (1.2 mg L^−1^) were reported to significantly enhance essential oil content and that of its components α‐pynene, β‐pynene, ρ‐pynene, α‐thujene, Calendula glycoside, α‐cadinene, cadinol, t‐muurolol, 1,8‐cineol and limonene in callus cultures of *Calendula officinalis* L. (marigold) in Murashige and Skoog (MS) medium supplemented with growth regulators 2,4‐D (2 mg L^−1^) and kinetin (0.2 mg L^−1^) [72]. In suspension cultures of *Aloe vera* L., AgNPs (0.625 mg ml^−1^) and TiO_2_ NPs (120 mg L^−1^) separately caused significant enhancement of aloin content after 48 h of exposure which gradually declined thereafter [73]. In *Capsicum frutescens* callus cultures in the presence of 2,4‐D and kinetin, AgNPs were demonstrated to cause a significant increase in capsaicin content compared with fruits of *Capsicum annuum* and *C. frutescens* [74]. Production of anti‐cancer compounds taxanes, taxol and baccatin III along with total soluble phenols, in cell suspension‐cultures of *Corylus avellana* L. (hazel) was enhanced about two‐fold by elicitation with 5 ppm AgNPs but declined at 10 ppm concentration [75]. TPC and anti‐oxidant capacity were significantly enhanced in *Vanilla planifolia* Jacks. ex Andrews (vanilla) shoots cultured in MS medium supplemented with 25 and 50 mg L^−1^ Ag NPs (35 ± 15 nm), but decreased at 100 mg L^−1^, an effect known as hormesis [76]. Cell suspension cultures of *Momordica charantia* L. (bitter gourd) amended with AgNPs (1–20 nm; 5 mg L^−1^) showed enhanced total phenolic and flavonoid contents compared with the control culture. There was also an increase in the contents of flavonols hydroxybenzoic and hydroxycinnamic acids which could be correlated with enhanced pharmacological activities (anti‐oxidant, anti‐diabetic, anti‐bacterial, anti‐fungal and anti‐cancer) of the plant [77]. Elicitation with AgNPs (45 mg L^−1^) was found to enhance stevioside production to the maximum in callus cultures of *Stevia rebaudiana* L. [78]. In cultures of the endangered medicinal plant *Caralluma tuberculata* (Asclepiadaceae), AgNPs (size 40 nm, concentration 60 μg L^−1^), when combined with plant growth regulators in MS media, was found to enhance callus biomass. At AgNP concentration of 90 μg L^−1^, the callus cultures showed higher production of phenolics (TPC: 3.0 mg), flavonoids (TFC: 1.8 mg), PAL activity (PAL: 5.8 U/mg) and anti‐oxidant activity (90%), respectively. At 90 μg/L AgNP concentration without growth regulators, enhanced activities of anti‐oxidant enzymes such as SOD, peroxidase, catalase and APX were observed [79].

In callus cultures of *Prunella vulgaris* L. (self‐heal), AgNPs and AuNPs alone (30 μg L^−1^) or in combination in different proportions, in presence of NAA (2 mg L^−1^) enhanced secondary metabolite production. The Ag–Au NPs (1:3) in combination with NAA induced maximum production of phenolics and flavonoids. Moreover, Ag–Au NPs (3:1) without NAA enhanced anti‐oxidant activity (87.85%) while AgAu (1:3) and AuNPs alone enhanced SOD and peroxidase enzymes to the maximum extent [80]. It was reported that the addition of 50 and 10 mg dm^−3^ nanocolloids of Ag (27.5 ± 4.8 nm) and Au (24.2 ± 2.4 nm) to cultures of *Lavandula angustifolia* (lavender) altered the essential oil composition. There was a decrease in lower molecular weight compounds (e.g. α‐pinene and β‐pinene, camphene, δ‐3‐carene, *p*‐cymene, 1,8‐cineole, *trans*‐pinocarveol and camphoriborneol), which were substituted by higher molecular weight compounds [τ‐cadinol 9‐cedranone and α‐cadinol 9‐cedranone, cadalene, α‐bisabolol, *cis*‐14‐nor‐muurol‐5‐en‐4‐one, (E,E)‐farnesol] [81].

Application of *copper nanoparticles* (∼1–2.7 nm; 0.5 mg L^−1^) and *cobalt nanoparticles* (∼1.3–3 nm; 0.8 mg L^−1^) during clonal micropropagation of *Mentha longifolia* increased the essential oil content by 2.226% and 2.19%, respectively [82]. In *in vitro* cultures of *Stevia rebaudiana* Bert. elicitation with CuO NPs (∼47 nm in diameter) caused a significant rise of bioactive major steviol glycosides (rebaudioside A and stevioside) at 10 mg L^−1^ concentration accompanied by an increase in total phenolic and flavonoid contents as well as anti‐oxidant activity [83]. CuO NPs (concentration 50, 100 and 150 mg L^−1^) were found to enhance phenolic and malonaldehyde content in *Solanum nigrum* L. callus cultures while upregulating the activities of anti‐oxidant defence enzymes peroxidase (POD), PPO and PAL [84]. In cell suspension cultures of the medicinal plant *Gymnema sylvestre* (Retz.) R. Br amended with CuO NPs (size 25–55 nm) at a concentration of 3 mg L^−1^ there was a nine‐fold increase in the production of gymnemic acid II and phenolic compounds compared with control [85].

*Aluminium oxide nanoparticles* (Al_2_O_3_ NPs) (concentration 10–100 μg L^−1^), when added to cell suspension cultures of *Nicotiana tabacum* L. (tobacco) were reported to significantly increase the phenolic content in a dose‐dependent and time‐dependent manner [86].

*Cobalt nanoparticles* (10 nm), when added to cell suspension cultures of the anti‐malarial medicinal plant *artemisia annua,* significantly enhanced artemisinin production (2.25‐fold at 5 mg L^−1^ after 24 h) while the expression of two artemisinin biosynthesis genes *SQS* and *DBR2* were downregulated [87].

Elicitation of shoot tip cultures of *Atropa belladonna* (deadly nightshade) with manganese trioxide (Mn_2_O_3_) NPs enhanced phenolics, flavonoids and alkaloids in a dose‐dependent manner while activating anti‐oxidant defence enzymes SOD, peroxidase, catalase and ascorbate peroxidase [88].

Exposure of suspension cultures of the Iranian medicinal herb *Dracocephalum polychaetum* Bornm. to *magnetite* (Fe_2_O_3_) NPs (100 ppm) along with static magnetic field (30 mT) was found to significantly enhance the contents of total phenolics, flavonoids, anthocyanins, lignin and malondialdehyde while increasing the activities of the enzymes PPO (which oxidises phenol) and PAL. There was an increase in the content and liberation rate of medicinal compounds such as RA, naringin, apigenin, thymol, carvacrol, quercetin and rutin [89].

The application of *TiO*_*2*_ *NPs* (4.5 or 6.0 mg L^−1^) significantly increased the content of gallic acid, chlorogenic acid, *o*‐coumaric acid, tannic acid and cinnamic acid in embryonic calli of *Cicer arietinum* (chickpea) [90] In callus cultures of *Hypericum perforatum,* addition of biologically synthesized *perlite NPs* (size 14.51–23.34 nm) and *TiO*_*2*_ *‐perlite* nanocomposites (size 15.5–24.61 nm) in the concentration range of 25–200 mg L^−1^, was reported to cause an increase in the content, variety and number of volatile compounds as well as in the amounts of hypericin and pseudohypercin [91].

*Zinc and iron oxide NPs* at 100 ppb concentrations significantly increased the production of hypericin and hyperforin in cell suspension cultures of *Hypericum perforatum* (St John's wort) [92]. Hormetic effect of *ZnO NPs* on *Lilium ledebourii* Bioss. cultures was reported where maximum content of phenolics and flavonoids was observed at ZnO NP concentrations of 75 and 25 mg L^−1^, respectively [93]. ZnO NPs (<100 nm in size; 500–1500 mg L^−1^) were also reported to increase phenolic and flavonoid production in callus cultures of *Brassica nigra* L. (Black mustard) in a concentration‐dependent manner while enhancing anti‐oxidant capacity [94]. In micropropagated shoots of *Stevia rebaudiana* Bert. ZnO NPs (size 34 nm) enhanced the production of steviol glycosides (rebaudioside A and stevioside) as well as total phenolic and flavonoid contents along with anti‐oxidant capacity at a concentration of 1 mg L^−1^, but the effect declined at higher concentration [95]. In suspension cultures of *Bacopa monnieri* (L.) Wettst., ZnO NPs (size 24 nm) at a concentration of 1 ppm were reported to cause a two‐fold increase in the content of the saponin bacoside A while lowering the expression of the *HMG CoA reductase* gene which controls the rate‐limiting step in bacoside A biosynthesis. It was suggested that ZnO NPs possibly have an effect on the isoprenoid pathway of biosynthesis [96]. Repeated elicitation of cell suspension cultures of *Linum usitatissimum* with ZnO NPs (size <35 nm, concentration 100 mg L^−1^) was reported to enhance the production of lignans in 15 days, and of phenolics, flavonoids and neolignans at 25 days [97]. ZnO NPs (size 34 nm) significantly enhanced thymol and carvacrol production in callus cultures of *Thymus* ssp., namely *T. vulgaris, T. daenensis* and *T. kotschyanus*. The highest increases for thymol and carvacrol were achieved with 150 mg L^−1^ of ZnO NPs in *T. kotschyanus* and *T. daenesis*, respectively [98].

*Bimetallic nanoalloys* of Cu and Au at 30 mg L^−1^ concentration were reported to stimulate biomass production and enhance the total content of phenolics and flavonoids as well as anti‐oxidant capacity in submerged adventitious root cultures of *Stevia rebaudiana* Bert. Maximum effect was seen with AuCu (1:3) NPs [99].

### 3.2 Carbon‐based nanoparticles

Supplementation of cultures of *Capsicum annuum* L. with *chitosan NPs* was found to enhance the contents of soluble phenols, proline and alkaloid while amplifying organogenesis through micropropagation like growth promoters, the most effective dose being 1 mg L^−1^. There was also an enhancement in the activities of the enzymes peroxidase, catalase and PAL [100].

In an interesting report *multi‐walled carbon nanotubes* (MWCNT) (5–15 nm in diameter) were found to enhance the content of secondary metabolites, namely phenolics, flavonoids, RA, caffeic acid and the activity of oxidative enzymes PPO, PAL and peroxidase (POD) in callus cultures of the medicinal plant *Satureja khuzestanica* in B5 medium, the most effective concentrations being 25 and 50 μg L^−1^ [101]. In seedlings of *Catharanthus roseus* (rose periwinkle), grown in MS basal medium, MWCNT treatment enhanced alkaloid and phenol contents 1.7‐fold and 23%, respectively, along with enhancement of activities of catalase, peroxidase and PAL and upregulation of the deacetylvindoline‐4‐*O*‐acetyltransferase (DAT) gene. This was accompanied by increase in plant growth indices like total biomass, leaf width, area and weight, root length, chlorophyll, carotenoid and protein contents and callus proliferation [102].

### 3.3 Nanoparticle‐mediated elicitation in hairy root cultures

Another strategy for the fast production of biomass with a high content of secondary metabolites from plant cultures involves the induction of hairy root cultures by *Agrobacterium rhizogenes*‐mediated genetic transformation, followed by elicitation with NPs. Elicitation with 900 mg L^−1^ Ag‐SiO_2_ core–shell NPs (average size 101.8 ± 8.9 nm) were reported to enhance Artemisinin production from 1.67 to 2.86 mg g^−1^ dry weight in hairy root cultures of *Artemisia annua* accompanied by an increase in H_2_O_2_ generation, lipid peroxidation and catalase activity [103]. Elicitation with AgNPs enhanced atropine production in hairy root cultures of *Datura metel* by 1.147‐fold, 1.117‐fold and 2.42‐fold in comparison to the control samples after 12, 24 and 48 h of treatment, respectively [104]. Hairy root cultures of *Hyoscyamus reticulatus* L. elicited with nano‐iron oxide (FeNPs) effectively enhanced the production of the tropane alkaloids hyoscyamine and scopolamine. The highest increase of hyoscyamine and scopolamine was observed with 900 mg L^−1^ FeNPs for 24 h and 450 mg L^−1^ FeNPs for 48 h, respectively [105]. Hairy root cultures of *Brassica rapa* ssp. *rapa* (turnip) elicited with AgNPs exhibited elevated levels of glucosinolates (glucoallysin, glucobrassicanapin, sinigrin, progoitrin, gluconapin, 4‐methoxy‐glucobrassicin, 4‐hydroxyglucobrassicin, gluco‐brassicin, neoglucobrassicin and gluconasturtin) and upregulation of their transcripts (*MYB34, MYB51, MYB28* and *MYB29*) [106]. In hairy root cultures of *Brassica rapa* ssp. *pekinensis* (Chinese cabbage), elicitation with CuO NPs was reported to significantly enhance the contents of glucosinolates (gluconasturtin, glucobrassicin, 4‐methoxyglucobrassicin, neoglucobrassicin, 4‐hydroxyglucobrassicin, glucoallysin, glucobrassicanapin, sinigrin, progoitrin and gluconapin) and upregulate the corresponding transcript (*MYB34*, *MYB122*, *MYB28* and *MYB29*) levels [107]. In both cases, there was an increase in total phenolic and flavonoid contents and upregulation of their gene expression (*PAL, CHI* and *FLS*). This was accompanied by enrichment of the phenolic compounds (flavonols, hydroxybenzoic and hydroxycinnamic acids). The effect on secondary metabolism could be correlated with increased anti‐oxidant, anti‐microbial and anti‐neoplastic activities of both the plants [106, 107]. In hairy root cultures of *Dracocephalum kotschyi* Boiss., elicitation with SiO_2_ NPs (100 mg L^−1^) resulted in an 8.26‐fold increase in the content of RA compared with control, after 48 h exposure time. Anti‐cancer flavonoids including xanthomicrol, cirsimaritin and isokaempferide increased 13‐fold, 13.42‐fold and 10‐fold, respectively, compared with the control. There was significant upregulation in the *pal* and RA synthase (*ras*) gene expressions [108]. Similar results were obtained using FeO NPs which gave a 9.7‐fold, 11.87‐fold, 3.85‐fold and 2.27‐fold enhancement in the contents of RA, xanthomicrol, cirsimaritin and isokaempferide, respectively, compared with controls, after 24 h of exposure to 75 mg L^−1^ Fe NP, along with the upregulation of *pal* and *ras* gene expressions under the influence of elicitation [109].

## 4 LIGAND HARVESTING OF PLANT SECONDARY METABOLITES USING NANOPARTICLES

**FIGURE 3:**
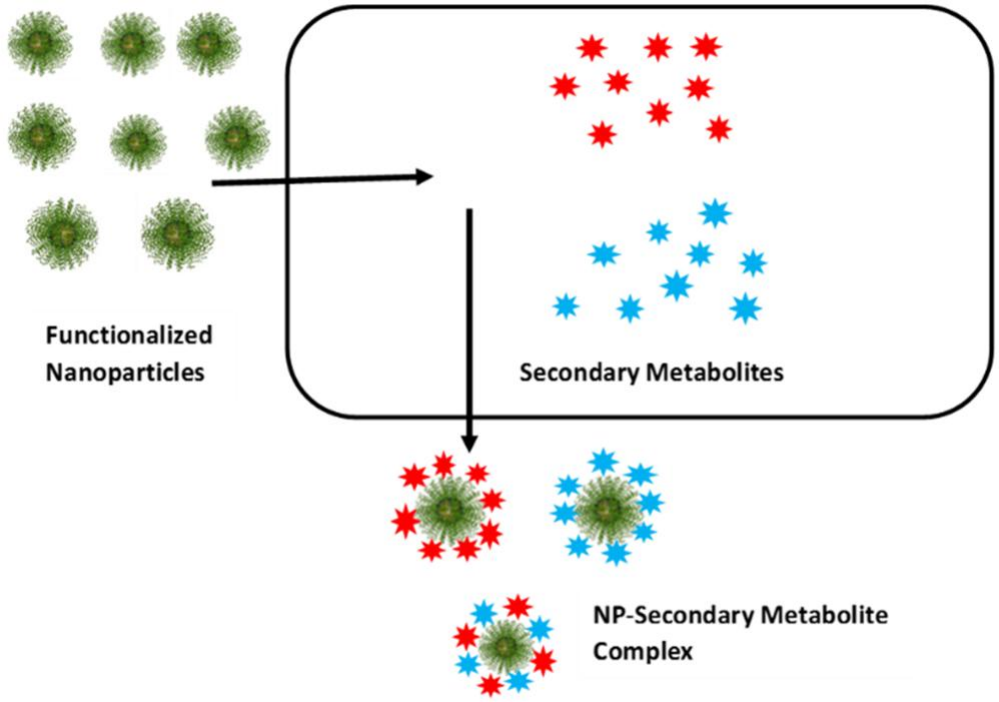
Schematic diagram of ligand‐harvesting of secondary metabolites from intact plant cells by functionalized nanoparticles

Interestingly, a novel application of NPs in secondary metabolite chemistry has opened up in the form of ligand fishing or ligand harvesting. Ligand fishing is an extraction technique based on the receptor theory. It is widely used to recover specific ligands from complex biological matrices using known or orphan receptors. The technique is widely used in protein purification. Nanoparticle‐mediated ligand fishing is particularly useful to screen and harvest specific bioactive compounds from complex botanical extracts. By this technique plant secondary metabolites may be nano‐harvested as conjugates directly from living plant cells using surface‐modified NPs, without damaging the host cells. The NPs, usually surface‐conjugated with specific receptors, enter living plant cells and are extracted after binding with targeted secondary metabolites, which may be subsequently separated and identified (Figure 3). The advantages of this technique is that it avoids the use of organic solvents for extraction, keeps host cells viable and also permits spectrometric identification of isolated compounds [110, 111]. Human serum albumin functionalized magnetic nanoparticles (HSA‐MNPs) (diameter ∼20 nm) coupled with electrospray ionization mass spectrometry have been employed for the fast extraction of four bioactive secondary metabolites progenin II, progenin III, dioscin and gracillin from herbal extracts of the Chinese medicinal plant *Dioscorea panthaica* [110]. Similarly, anatase TiO_2_ NPs (2.8 ± 1.4 nm in size) were used to harvest enediol and catechol‐rich flavonoids, particularly quercetin‐derivatives, from living cells of the plant *Arabidopsis thaliana* by forming flavonoid‐NP conjugates, without affecting the viability of the source plant [111]. This technology is still in its infancy and needs further exploration and investigation.

## 5 MECHANISM OF ACTION OF NANOPARTICLES ON PLANT SECONDARY METABOLISM

**FIGURE 4:**
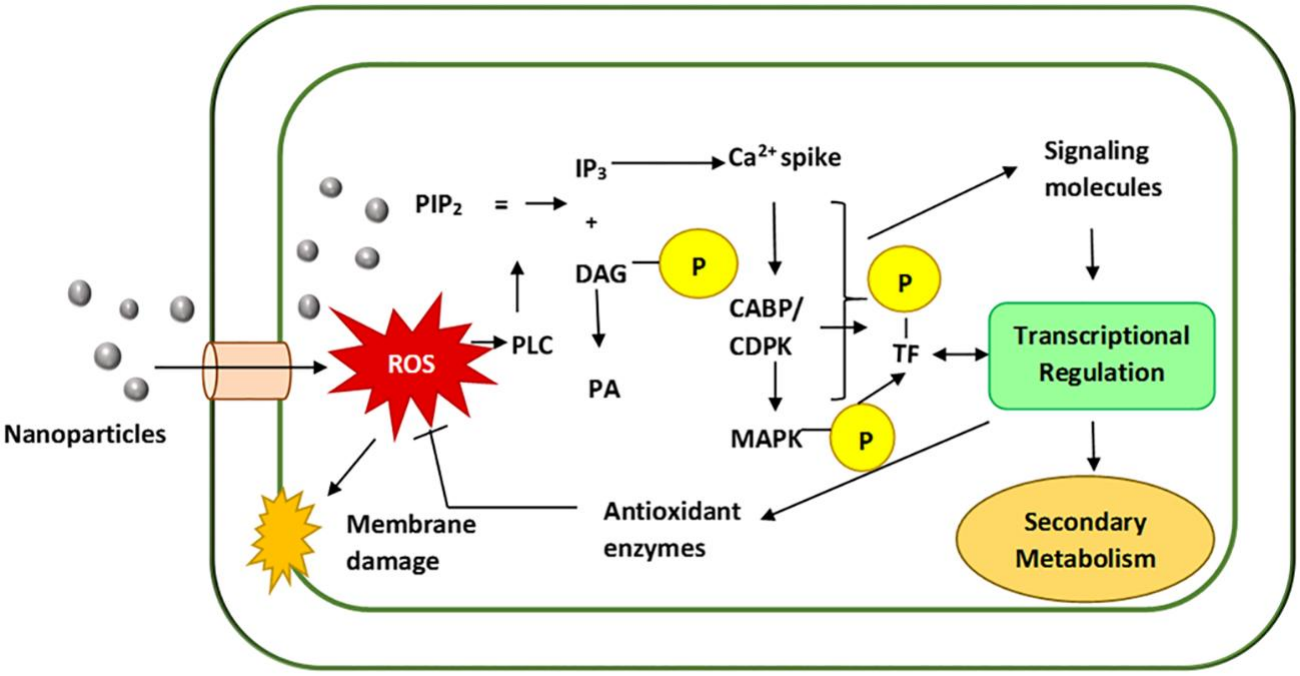
Schematic representation of the probable mechanism of modulation of plant secondary mechanism by nanoparticles

It must be reiterated that increased or *de novo* synthesis of secondary metabolites by plants is a response to abiotic and biotic stress and is a defence mechanism. A brief overview of the mechanism is presented here (Figure 4). Plant response to stress occurs both at the cellular as well as the organismic level. The stress signal is first perceived by the receptors present on the cell membrane. This activates a large and complex signalling cascade intracellularly, including the generation of secondary signal molecules. The stress signal can first activate phospholipase C (PLC), which hydrolyses phosphatidyl‐inositol 4,5 bisphosphate (PIP_2_) to generate inositol triphosphate (IP_3_) and diacylglycerol (DAG). IP_3_ diffuses into the cytosol and subsequently releases of Ca^2+^ ions from intracellular Ca^2+^ stores, resulting in an increase in the level of Ca^2+^ ions in the cytosol. Ca^2+^ release also occurs primarily from extracellular source (apoplastic space). The increased Ca^2+^ concentration is sensed by calcium‐binding proteins (CaBP, calcium sensor) such as calmodulin (CaM), calmodulin‐like proteins (CML), phospholipase D, annexins, calreticulin, calnexin and Pistil‐expressed Ca^2+^ binding protein (PCP) or directly by calcium‐dependent protein kinases (CDPK). These sensors recognize and decode the information provided in the calcium signatures, relay the information downstream to initiate a phosphorylation cascade leading to regulation of gene expression. Several reports suggest that Ca^2+^ regulates the transcription of target genes by altering the phosphorylation status of specific transcription factors (TF). DAG is phosphorylated by DAG kinase to give phosphatidic acid (PA), another signalling molecule. Various other chemical signals including abscisic acid (ABA), salicylic acid (SA), polyamines, jasmonates (JA) and nitric oxide are involved in stress responses in plants, often through cross‐talk [112, 113]. The biosynthesis of many of the secondary metabolites is mediated through (methyl) jasmonate [(Me)JA)], a plant hormone produced in response to stress. Production of secondary metabolites is controlled at the level of expression of the biosynthetic genes by developmental and tissue‐specific factors or by external signals [114, 115, 116]. In the resting state, a family of proteins called JAZ interacts and repress certain downstream TF (e.g. MYC2) to suppress JA responses. In response to JA signal, the F‐box protein COI1 interacts with and ubiquitinates JAZs tagging them for degradation through the 26S proteasome, thereby releasing downstream TFs to regulate gene expression and activate JA responses [117].

Nanoparticles are known to cause a certain degree of phytotoxicity, especially at high concentrations [9]. NPs, depending on size, have a high degree of cell penetrability. They can enter the plant cell through the apoplast and cross the plasma membrane by endocytosis; subsequently, they can be translocated within the plant by the symplastic flow [118]. There is also evidence of transport of NPs into subcellular organelles like nucleus, plastids and vacuoles [119, 120]. NPs can interfere with electron transport chain in chloroplasts and mitochondria causing oxidative burst and accumulation of reactive oxidative species (ROS) like hydrogen peroxide (H_2_O_2_), superoxide anions (O_2_^**.**¯^), hydroxyl radical (OH^**.**^) and singlet oxygen (^1^O_2_). Induction of reactive nitrogen species (RNS) (NO nitric oxide), due to exposure of duckweed *Spirodela punctata* to Ag and ZnO NPs [121] and of cultured tobacco BY cells to Al_2_O_3_ NPs [86] have also been reported. NO is known to be an important elicitor of plant secondary metabolism [122]. Metal and metal oxide NPs have been extensively studied for their toxic effects on plants which are thought to largely occur through (ROS) burst [10]. ROS are known to interact with almost all cellular components causing protein modifications, lipid peroxidation and DNA damage [123]. They also activate the plant's enzymatic and non‐enzymatic anti‐oxidant system. The key enzymes involved in anti‐oxidant defence response are SOD that catalyses the conversion of O_2_^**.**¯^ to either molecular oxygen (O_2_) or H_2_O_2_, APX that detoxifies H_2_O_2_ using ascorbic acid as a substrate, catalase which decomposes H_2_O_2_ to water and O_2_ and glutathione‐S‐transferases (GST) which catalyse the conjugation of the reduced form of glutathione (GSH) to xenobiotic substrates for the purpose of detoxification.

Nanoparticles may enter plant cells through membrane receptors or plasmodesmata causing reactive oxygen species (ROS) burst which may damage the plasma membrane. The ROS activates Phospholipase C (PLC) which hydrolyses phosphatidyl‐inositol 4,5 bisphosphate (PIP_2_) to generate inositol triphosphate (IP_3_) and diacylglycerol (DAG). DAG is phosphorylated by DAG kinase to give phosphatidic acid (PA), a signalling molecule. IP_3_ diffuses into the cytosol and subsequently causes a Ca^2+^ ion spike. The increased Ca^2+^ concentration is sensed by calcium‐binding proteins (CaBP, calcium sensor) or directly by calcium‐dependent protein kinases (CDPK), which recognize and decode the information provided in the calcium signatures, relay the information downstream to initiate a phosphorylation cascade, including the upregulation of mitogen‐activated protein kinase (MAPK) cascades. Various other chemical signals including abscissic acid (ABA), salicylic acid (SA), polyamines, jasmonates (JA) and nitric oxide are involved in stress responses often through cross‐talk. Ca^2+^ possibly regulates the transcription of target genes by altering the phosphorylation status of specific transcription factors (TF). MAPK phosphorylation and activation of downstream transcription factors like WRKY generally lead to the transcriptional upregulation of secondary metabolism as well as activation of anti‐oxidant defence enzymes.

The enzymatic anti‐oxidant defence also involves the downregulation of dehydroascorbate reductase (DHAR) and monodehydroascorbate reductase (MDAR) enzymes that regulate the cellular Asc redox state [124]. Depending on the delicate balance between ROS generation and scavenging, ROS may cause oxidative damage or act as cellular signalling molecules. NADPH oxidases are known to act as key signalling nodes in ROS regulation network of plants integrating numerous signal transduction pathways and mediating multiple biological processes. ROS are also thought to modulate secondary metabolism either directly or by acting as signals for other inducers like JA, SA, ethylene, NO and brassinosteroids. ROS generation is thought to result in cytoplasmic Ca^2+^ spike resulting in upregulation of MAPK cascades similar to other abiotic stresses [11, 125]. NPs either mimic Ca^2+^ or signalling molecules in the cytosol to be sensed by calcium‐binding proteins (CaBPs) or other NP‐specific proteins [126]. MAPK phosphorylation and activation of downstream TF like WRKY generally lead to the transcriptional upregulation of secondary metabolism in plants [127, 128, 129].

## 6 PERSPECTIVES

The role of nanoparticles as inducers of abiotic stress and toxic effects in plants has been understood for a considerable length of time. However, it is only recently that they are being regarded as tools for molecular pharming to elicit beneficial secondary metabolites in plants both *in vivo* and *in vitro* and also as agents for nano‐harvesting secondary metabolites from plant cells as conjugates. Both these applications have immense commercial potential. The function of nanoparticles as elicitors of secondary metabolites is greatly dependent on their chemical and mineralogic composition, size, sometimes shape and also on the concentration of application. As mentioned earlier, the effect of NP concentration appears to be hormetic, as shown in *Bacopa monnieri* (L.) Pennell [51] and *Vanilla planifolia* Jacks. ex Andrews [76]. According to the report of Syu et al. [130], the shape of nanoparticles plays a significant role in the elicitation of anthocyanins in *Arabidopsis* seedlings, the most effective being spherical NPs. Since each nanoparticle‐plant system is unique, it would be necessary to determine the optimum size, shape and other parameters as well as concentration of NPs for maximum production of secondary metabolites on a case‐by‐case basis. Also, the physical and chemical properties as well as biological activities of the secondary metabolites obtained from plants treated with NPs both *in vivo* and *in vitro*, should be studied in detail to determine their quality and efficacy. The toxicological risks associated with the application of nanoparticles for the purpose of secondary metabolite elicitation must also be evaluated and safety standards formulated vis‐à‐vis the dose and route of administration for each plant. Stringent dose‐response studies must be undertaken to determine the optimum concentration for each NP‐plant system for maximum secondary metabolite yield with minimum toxic effects on the plant as well on as the consumer of the product and the environment. The entire life cycle of these NPs should be monitored, including their fabrication, storage and transportation, application and potential abuse and disposal. The penetration, translocation and bioaccumulation process in each nanoparticle‐plant system must be investigated on a case‐by‐case basis [4]. Moreover, most of the results reported are from laboratory‐scale or at the most, greenhouse experiments. The feasibility of applying this technology for scaled‐up production in field conditions or industrial set‐ups remains to be evaluated along with the costs of such commercial production. It is likely that the NPs of noble metals like Ag, Au and Cu may too expensive to apply on a commercial scale and less expensive alternatives like that of Al, Fe, Si, Zn or even chitosan may be preferable. In conclusion, it may be stated that NP‐mediated elicitation and extraction of plant secondary metabolites both *in vivo* and *in vitro*, holds the prospect of positively impacting industrial activities utilizing secondary metabolites, if the technology is standardized and adapted for commercial application.
